# Comprehensive multi-modality treatment of thoracic aorta pseudoaneurysms: a single-center experience

**DOI:** 10.1007/s11748-023-01986-9

**Published:** 2023-11-25

**Authors:** Sandra Recicarova, Michael Jonak, Ivan Netuka

**Affiliations:** 1 Department of Cardiovascular Surgery, Institute for Clinical and Experimental Medicine (IKEM), Videnska 1958/9, Prague, 140 00 Czech Republic; 2 First Faculty of Medicine, Charles University, Prague, Czech Republic; 3 Second Department of Surgery, Department of Cardiovascular Surgery, First Faculty of Medicine, Charles University, Prague, Czech Republic

**Keywords:** Aortic pseudoaneurysm, Aortic dissection, Bentall surgery, Endoluminal graft implantation, Septal occluder implantation

## Abstract

**Introduction:**

Thoracic aorta false aneurysms (TAFA) are unexplored complications after cardiac surgery associated with significant morbidity and mortality. Therefore, the purpose of this study was to examine the clinical profiles, surgical techniques, and operative outcomes, of patients treated for TAFA at a single institution.

**Methods:**

From 1996 to 2022, 112 patients were treated for aortic pseudoaneurysm (mean age 55 ± 14 years, 78 patients were male). In the majority of the patients (90%) TAFA developed after previous cardiovascular surgery, the most common diagnosis and surgical procedure preceding the TAFA development was an aortic dissection (52%) and Bentall procedure (47%). In the rest of the cohort, the leading cause was trauma.

**Results:**

Sixty-one percent of patients were indicated for reintervention (surgical reoperation, endoluminal graft implantation, septal occluder implantation, coil embolization, or a combination of procedures). Overall, 52 patients had undergone cardiac reoperation. TAFA was resected and the aorta was repaired in 55% or replaced in 45%. Operative mortality was 5.7%. In postoperative follow-up, a hypoechogenic lesion encircling aortic prosthesis was present in 94%, therefore it was determined as a negative prognostic factor. The mean follow-up was 13.2 ± 19.4 years.

**Conclusion:**

Although there is no specific approach how to prevent TAFA development, maintaining normal blood pressure and regular follow-up should be applied. More frequent follow-ups should be performed in patients with a hypoechogenic lesion encircling and aortic prosthesis. Early detection during long-term postoperative follow-up, an individually tailored approach of a multidisciplinary team is necessary for favorable treatment outcomes.

## Introduction

Thoracic aorta false aneurysms (TAFA) can be a serious complication of aortic surgery. These aneurysms are caused by a disruption in the aortic wall, which can lead to blood leaking out and being contained by nearby structures. Although rare, they can be life-threatening and often develop from cannulation or clamping sites, aortotomy sites, or graft/coronary reimplantation sites [1]. The development of these aneurysms is usually asymptomatic, but they can expand over time and cause chest pain, coronary compression, dysphagia, or stridor [2].

It's important to evaluate every case thoroughly and develop a treatment strategy that is tailored to the individual to avoid perioperative death. The multidisciplinary team will determine whether the patient is indicated for surgical reoperation, endoluminal graft implantation, septal occluder implantation, or coil embolization. If the patient has small, stable TAFA or is unacceptable for reoperation and endovascular repair, conservative treatment will be established [3].

We conducted a thorough review of TAFA patients who underwent surgery at our institution. We focused on analyzing clinical presentation, etiologies, genetic screening, diagnostic methods, surgical techniques, and long-term results to develop a comprehensive understanding of this condition. To ensure the best possible outcome, we tailored our treatment strategy to each individual patient, taking into account factors such as their overall health and the severity of their condition. Our primary goal is to ensure that every patient received safe, effective care to avoid any perioperative complications.

## Patients and methods

At the Institute for Clinical and Experimental Medicine in Prague, Czech Republic, we reviewed a group of 112 patients who were treated for pseudoaneurysm of the thoracic aorta between January 1996 and December 2022. Our institution carries out between 80 to 120 surgeries on the thoracic aorta annually. We ensure that all patients receive follow-up care after their surgery, which enables us to identify any complications that may arise following aortic surgery.

Preoperative, operative, and postoperative data were obtained from the Cardiovascular Information Registry in the Czech Republic. General systematic follow-up was obtained through echocardiographic or computed tomography assessment at our institution. Other parts of follow-up were reported from referring cardiologists and systematic formal interviews with patients and family members.

The Kaplan Meier method and the Log-Rank test were used to present and compare survival estimates for patients with aortic aneurysms, aortic dissections, and other diagnoses (software JMP 15.2.0, 2019 SAS Institute, NC, USA).

The institutional ethics committee waived the need for informed consent as the study was designed as retrospective.

## Results

In our study, we found that false aneurysms were identified in 112 patients during 26 years of follow-up. The baseline characteristics are presented in Table 1. The majority of patients were men, and the mean age at the time of surgery was 55 ± 14 years. We also noted that 26 patients suffered from obesity with a BMI of over 30 kg/m^2^, but only one patient was malnourished.

**Table 1:** Preoperative demographic characteristics

Total number of patients	112
Mean age at the time of surgery (years)	55 ± 14
Male	78 (69.6%)
Female	34 (30.4%)
Obesity (BMI > 30)	26 (23.2%)
Denutrition (BMI < 20)	1 (0.9%)
Connective tissue disorder	13 (11.6%)
Hypertension	89 (79.5%)
Diabetes mellitus	7 (6.3%)
Atherosclerosis	20 (17.9%)
Stroke / neurological disorder	29 (25.9%)
Renal failure	9 (8.0%)
Atrial fibrillation	43 (38.4%)
Immunosuppression	4 (3.6%)
*Aortic valve disease*	
Aortic regurgitation	78 (69.6%)
Aortic stenosis	7 (6.3%)
Mixed aortic valve disease	4 (3.6%)
Without aortic valve disease	23 (20.5%)

Thirteen patients were diagnosed with connective tissue disorder (10 patients with Marfan syndrome, 1 patient with Loyes-Dietz syndrome, and 2 patients with unidentified connective tissue disorders). The majority of patients, 89 in total, suffered from hypertension. Of the other comorbidities we observed, 7 patients had diabetes mellitus, 20 patients had atherosclerosis, and 29 patients had suffered from stroke or other neurological disorders. Four patients were treated with immunosuppression therapy, and 43 patients experienced atrial fibrillation.

Our study found that aortic regurgitation was the most common aortic valve disorder in 78 patients, while aortic stenosis was observed in only seven patients. Interestingly, although infected tissue is considered a predisposing factor for TAFA development [4], only four of our patients were treated for infection at the time of TAFA detection (Table 1).

It is interesting to note that the majority of TAFA cases (90%) developed after previous cardiovascular surgery, while only 10% were a result of trauma, such as car accidents. The primary indication for surgery in these cases was dissection (52%), followed by aortic aneurysm (31%), intramural hematoma, rupture, or trauma (15%), and coarctation (2%).

A total of 101 patients had previous cardiac surgery (89 had one previous cardiac operation, eight patients had two, and four patients had three previous cardiac surgeries). Sternotomy was performed in every case. Nine patients were operated on in different cardiovascular centers. Eighty-four patients had undergone a single procedure, and 17 patients had undergone a combined procedure. As demonstrated in Table 2, the most common procedure that led to the development of TAFA was the Bentall procedure (47%), ascending aorta replacement (12%), and aortic valve resuspension with ascending aorta replacement (9%). One patient had undergone heart transplantation (Table 2).

**Table 2:** Previous surgery characteristics

Surgery	120 procedures in 101 patients (84 single, 17 combined procedures)	%
Aortic valve resuspension + ascending aorta replacement	9	7.5
Aortic valve resuspension + ascending aorta replacement—hemiarch	8	6.7
Aortic valve replacement	1	0.8
Aortic valve + ascending aorta replacement	6	5.0
Aortic valve + ascending aorta replacement with homograft	1	0.8
Bentall	47	39.2
Bentall—hemiarch	4	3.3
Bentall + arch replacement	1	0.8
Ascending aorta replacement	12	10.0
Ascending aorta replacement—hemiarch	2	1.7
Ascending aorta + arch replacement	2	1.7
Ascending aorta patch	3	2.5
Heart transplant	1	0.8
Aortic coarctation	2	1.7
Different concomitant procedures	10	8.3
Coronary artery bypass graft	11	9.2

As described in Table 3, the mean cardiopulmonary bypass time was 173 ± 54 min, the mean aortic clamping time was 110 ± 32 min, and the mean circulatory arrest time was 18 ± 28 min. A proximal suture was stabilized with PTFE—pledget in 22 patients (from the intraluminal part in one patient, from the external part in 18 patients, and both parts of the suture in 3 patients). The distal suture was stabilized with PTFE—pledget in 37 patients (from the external part in 27 patients, and both parts of the suture in 10 patients). After trying to stabilize the anastomosis with PTFE-pledget to prevent TAFA, it was disappointing to find out that it did not have the intended effect as it was an improvement that would decrease the incidence of aortic pseudoaneurysms.

**Table 3:** Peri and postprocedural characteristics

Duration of CPB (min)	173 ± 54
Cross-clamp time (min)	110 ± 32
Duration of circulatory arrest (min)	18 ± 28
Proximal suture with PTFE—pledget	22 (19.6%)
Distal suture with PTFE—pledget	37 (33.0%)
Use of biological glue	78 (69.6%)
Revision surgery	17 (15.2%)
Wound infection	8 (7.1%)
Multiple surgeries until TAFA development	12 (10.7%)
Antiplatelet therapy after surgery	22 (19.6%)
Anticoagulation after surgery	74 (66.1%)

Biological glue was used in a majority of the cases 78% (GRF glue in 40 cases, Bioglue in 13 cases, Tisseal in 3 cases, or a different combination of them in 22 cases). While some researchers suggest that biological glue may increase the risk of aortic pseudoaneurysm, we find it essential for hemostasis during surgery and therefore we did not try to decrease its use.

Surgical re-exploration was required in 17%. Eight patients had wound infections. Three patients had deep sternal wound infections requiring extensive debridement. Postoperatively, 74% were indicated for anticoagulation and 22% for antiplatelet therapy (Table 3).

The median interval between the primary operation and the detection of the lesion was 6.4 ± 6.6 years. The median interval between the detection of the lesion and reintervention was 1.6 ± 2.3 years. The mean follow-up was 13.2 ± 19.4 years. The mean TAFA diameter was 52 ± 28 mm (range 10 to 150mm).

The predominant symptoms reported by patients were chest pain (12 patients), dyspnea (7 patients), acute heart failure (3 patients), hoarseness (2 patients), and vena cava superior syndrome (one patient). Seventy-five percent of patients were asymptomatic. The initial diagnosis was made by computed tomography and completed with an echocardiogram to evaluate every case correctly.

The most frequent disruption sites were proximal anastomosis (21%), distal anastomosis (18%), left coronary artery reimplantation (11%), and right coronary reimplantation site (7%). Other localization such as the central anastomosis of coronary bypass, the site behind the left subclavian artery, and the aortic coarctation were present in 21%. Multiple disruption sites developed in 34%.

Based on the postoperative follow-up study, it was found that a hypoechogenic lesion encircling the aortic prosthesis on CT scans was a negative prognostic factor in 94% of cases. This factor determination excluded 11 patients after trauma and 2 patients after surgery for aortic coarctation from a cohort of 112 patients. Out of 99 patients after cardiac surgery, 39 had the hypoechogenic lesion with a maximum size of 1cm, and 38 had a lesion greater than 1cm that persisted in the following scans. Only five patients did not have the lesion, and in 17 patients, lesion presence could not be confirmed due to the lack of digitalization and data archiving during the initial surgery and CT scans. It seems that the size of the lesion may be correlated with an increased risk of developing TAFA. However, it's important to note that this lesion has not been observed during surgery, so we cannot confirm if it is a seroma.

**Fig. 1:**
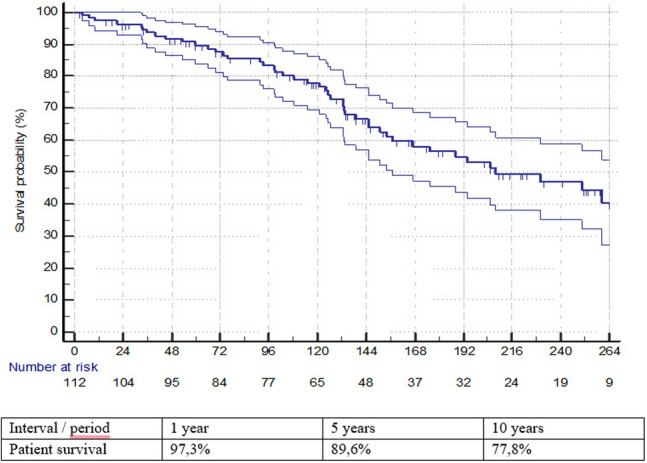
Kaplan–Meier analysis of survival of patient’s cohort with TAFA

**Fig. 2:**
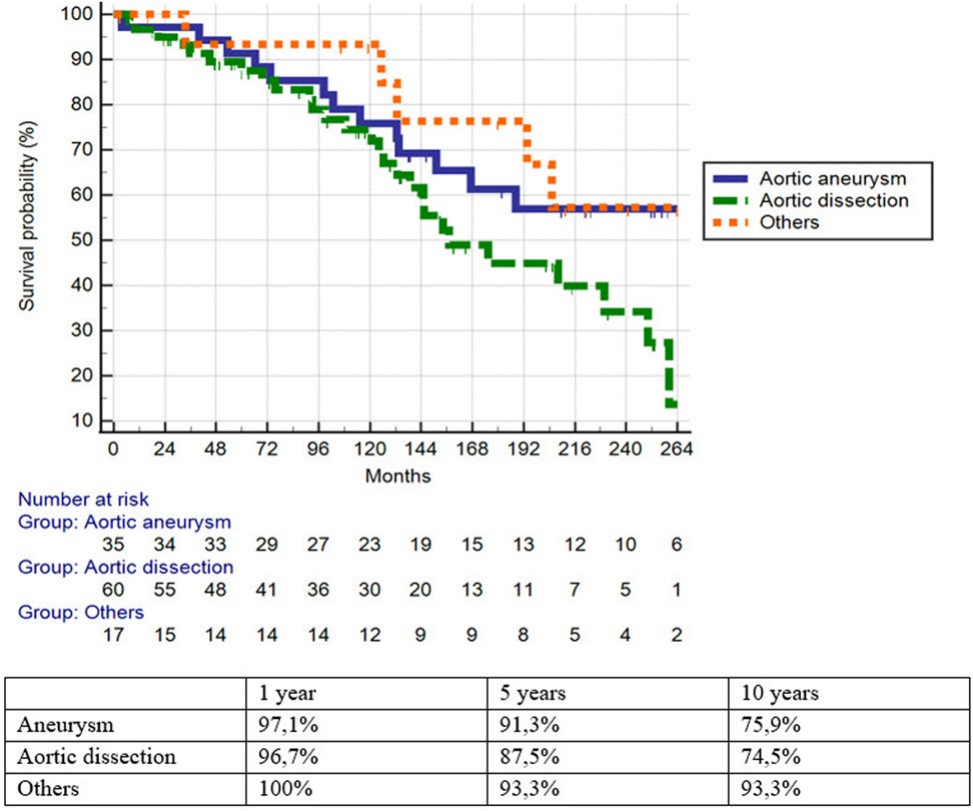
Analysis of survival probability of patients with TAFA that occurred after repair of aortic aneurysm, aortic dissection, or other diagnoses

One year patient survival was 97.3% (patients with aortic aneurysm 97.1%, aortic dissection 96.7%, other diagnosis 100%), 5 years patient survival was 89.6% (patients with aortic aneurysm 91.3%, aortic dissection 87.5%, other diagnosis 93.3%) and 10 years patient survival was 77.8% (patients with aortic aneurysm 75.9%, aortic dissection 74.5%, other diagnosis 93.3%) (Figs. 1, 2). The differences in survival rates between patients with aortic aneurysms, aortic dissection, and other diagnoses were insignificant (p = 0.0871).

## Surgical technique

**Fig. 3:**
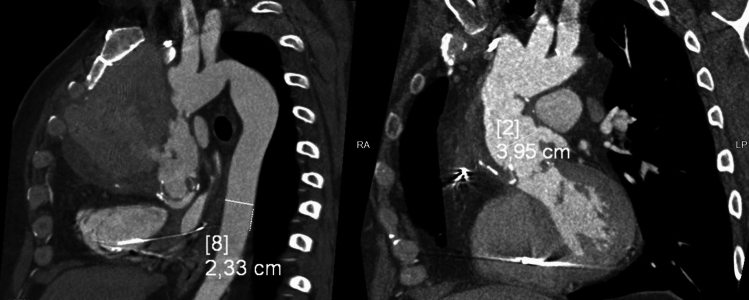
CT scan before and after reoperation

**Fig. 4:**
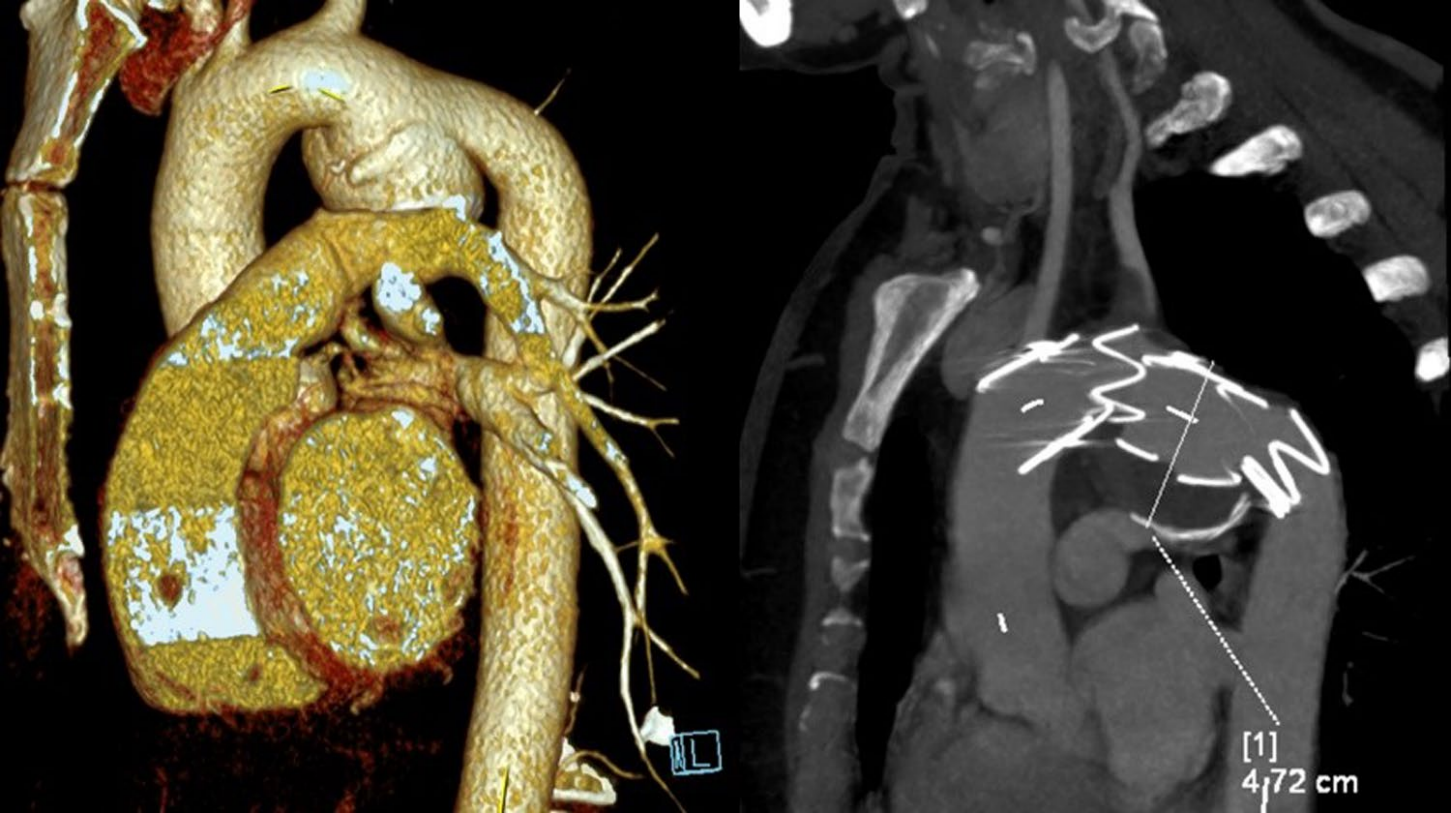
CT scan of aortic pseudoaneurysm caused by trauma and after stentgraft implantation

**Fig. 5:**
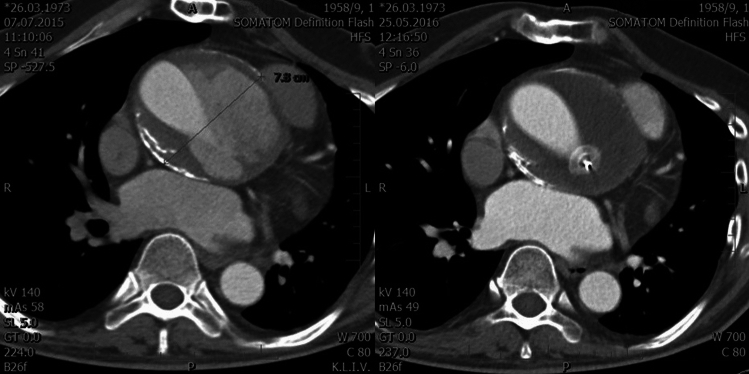
CT scan before and after occluder implantation

The majority of the patients (68 subjects, 61%) were indicated for reintervention, with an open reoperation being performed in 39 cases (Fig. 3). A combination of reoperation with endoluminal graft implantation was used in 7 cases; a combination of reoperation with septal occluder implantation in 4 cases; and a combination of reoperation with endoluminal graft and with septal occluder implantation in 2 cases. Ten patients were indicated for endoluminal graft implantation (Fig. 4), five patients for septal occluder implantation (Fig. 5), and one patient for coil embolization. Forty-four patients either declined reintervention or were deemed unsuitable for a redo procedure and were subsequently entered into a surveillance program.

Throughout the surgery, patients received careful monitoring using transesophageal echocardiography. The surgical approach was determined based on a variety of factors, including the size and location of the TAFA, the presence of pseudoaneurysm underneath the sternum, the presence of infection, any valvular diseases, and any associated cardiac problems that required concurrent surgical treatment. In every instance, a median sternotomy was performed to ensure the best possible outcome for each patient. These measures highlight the commitment to personalized and precise care that is crucial for achieving the best results.

The arterial cannulation site was the femoral artery in 39%, the aorta or brachiocephalic trunk in 33%, and the axillary or subclavian artery in 28%. The venous cannulation site was the right atrium in 46%, the femoral vein in 38%, and the vena cava superior and inferior in 16%.

In eight patients, cardiopulmonary bypass was started before resternotomy due to the potential risks involved in opening the chest near the pseudoaneurysm. For four patients, this led to deep hypothermic circulatory arrest, and resternotomy was only performed once their body temperature had reached 18 °C.

The mean cardiopulmonary bypass time was 206 ± 137 min, and the mean aortic clamping time was 114 ± 62 min. A deep hypothermic circulatory arrest was used in 34% and the meantime was 46 ± 38 min. Antegrade cerebral perfusion was used in 19%.

In the treatment of pseudoaneurysms, the method of closure used depended on the size of the defect and the absence of infection. For small defects, a suture was primarily used to close the segment of the aorta where the pseudoaneurysm was located in 14% of cases. However, for larger defects, the aortic repair was performed after debriding the pseudoaneurysm sac with interrupted pledget-supported sutures in 41% of cases. Surgeons used either PTFE-pledget (34%) or bovine pericardium (7%), depending on their experience.

Replacement with a tube graft (45%) was performed in patients with large TAFA, mediastinitis or graft infection, associated prosthetic aortic valve endocarditis, or impossibility of repair taking into account the risk of recurrence.

In cases where TAFA occurs from the coronary artery reimplantation site, the best course of action was to mobilize the coronary ostia out of the surrounding aorta and reimplant the coronary buttons with continuous 5–0 polypropylene suture in 60% of cases. If sacrificing this repair is necessary to achieve a secure inflow suture line, then a saphenous vein graft was interposed to the distal coronary artery in 40% of cases.

In cases where surgical repair was not an option for TAFA, debranching, and endoluminal graft implantation were performed in three patients, or carotid-subclavian bypass implantation was performed in one patient. It's reassuring to know that there are alternative procedures available.

Concomitant surgery included aortic valve replacement in five patients, mitral valve replacement in two patients, coronary artery bypass grafting in one patient, or multiple combined procedures in four patients.

In four patients resternotomy caused profuse hemorrhage due to opening TAFA. Perfusion flow on cardiopulmonary bypass was immediately reduced and the head was tilted down to prevent cerebral embolism. A Foley balloon was inserted into the defect which allowed to separate sternal edges and to identify the native aorta. In two patients the pump was temporarily arrested and repair was performed during hypothermic circulatory arrest. In one patient profuse hemorrhage occurred during dissecting the heart from adhesions and in three patients it occurred after completing the surgery.

The most often complication was a neurological deficit in five patients, ventricular arrhythmias in two patients, heart failure in 2 patients, multiple organ dysfunction syndromes in one patient, renal insufficiency in one patient, and a thrombus in the coronary artery in one patient.

Out of 68 reinterventions for TAFA, recurrence occurred in 22 patients (19 patients after reoperation). In 12 patients TAFA occurred in the same disruption site as previously and in 10 patients TAFA occurred in another disruption site. The mean time to recurrence of TAFA was 2.8 ± 2.9 years. Four patients were indicated for septal occluder implantation and four patients were indicated for redo surgery.

The cause of death was found by reviewing clinical records and death certificates. Operative mortality was 5.7%. All three patients died of massive hemorrhage (two during the sternotomy and one after completing the surgery). One year patient survival was 97.3%, five years was 89.6% and ten years was 77.8%. During the follow-up four patients died of infectious complications, three died shortly before planned reoperation and the rest died of other comorbidities (i.e. cancer, neurological diseases) or new diagnosis (i.e. Covid-19, pulmonary embolism).

## Discussion

Over the past three decades, there have been numerous instances of mediastinal false aneurysms that have been reported by investigators. However, only a handful of studies with a sample size of over ten patients have been published. As per our understanding, this research study represents one of the largest cohorts of patients with TAFA [5].

Unlike in other studies [1, 9, 10, 13], a significant portion of our patients (75%) did not experience any symptoms, and their TAFA diagnosis was only revealed during routine follow-up. The median interval between their prior operation and the detection of the lesion was 6.4 ± 6.6 years, highlighting the need for long-term monitoring after aortic surgery. Our mean follow-up was 13.2 ± 19.4 years, which further emphasizes the importance of ongoing surveillance. For those with recognized risk factors such as previous aortic dissection, connective-tissue disorders, postoperative graft infection, or history of Bentall procedure, lifelong follow-up is mandatory [6].

It's worth noting that in the reported series, chest pain was the most common symptom associated with the progression of TAFA. This pain was often caused by compression of surrounding structures, aortic stretching, or cardiac ischemia [7]. Dyspnea was also present in cases where TAFA was causing compression of bronchi or if heart failure was occurring. Other symptoms included heart failure, which was related to valvular regurgitation, compression of surrounding structures, and cardiac compression, as well as hoarseness resulting from compression of the recurrent laryngeal nerve [8].

TAFA can result from a disruption of the aortic wall, with most cases (90%) occurring after previous cardiac operations and 10% following trauma. The most common locations for false aneurysms are proximal and distal ascending aortic anastomosis disruptions and coronary button reimplantation sites. Less common areas include the aortotomy site and saphenous vein graft anastomotic site. While leaks at the aortic cannulation site and aortic vent site have been reported, this type of pathology has not been observed by our team [9]. In cases of TAFA after trauma, the predominant site is behind the left subclavian artery.

**Fig. 6:**
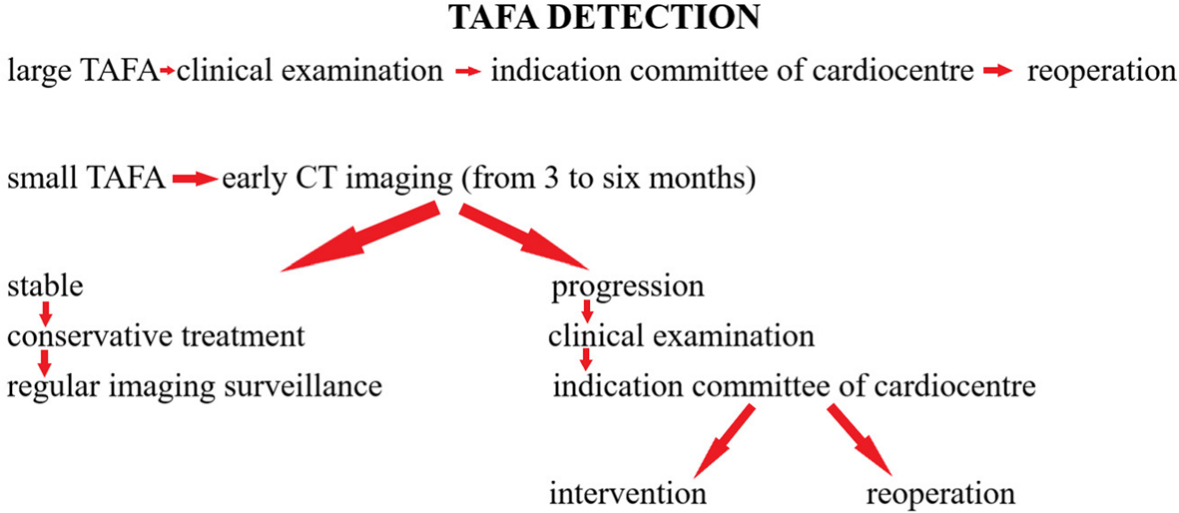
Diagnostic and therapeutic algorithm

Our institution offers a range of treatment options for patients with TAFA. We make sure that patients are fully informed about their medical condition and respect their decisions if they decline further intervention. Our multidisciplinary team carefully evaluates each case and recommends surgical reoperation, endoluminal graft implantation, septal occluder implantation, or coil embolization based on individual patient needs. In some cases, we may recommend conservative treatment for patients with small, stable TAFA or those who are not suitable candidates for reoperation or endovascular repair. The method of repair was individually tailored to each patient. Our diagnostic and therapeutic algorithm is described in Fig. 6.

When it comes to treating TAFA, a surgical approach is a complex treatment that requires thorough planning and consideration of various factors to ensure its success. We took into account the location of the TAFA, its proximity to the undersurface of the sternum, the disruption site, and any comorbidities when evaluating each case. Extramediastinal cannulation, hypothermic low-flow cardiopulmonary bypass, and deep hypothermic circulatory arrest are well-known strategies; and were used to prevent periprocedural exsanguination [10]. Median sternotomy was our incision of choice in every case. The majority of patients were cannulated via femoral vessels, or the aorta and the right atrium. Reoperations for false aneurysms must provide secure aortic repair. Depending on the size and location of the neck, we used a simple graft, composite replacement, patch, or simple suture [11].

For patients who are in critical condition preoperatively, the chance to avoid open surgery is quite appealing. While there have been some great advancements in endovascular techniques, surgery is still considered the best treatment option. Depending on the location of the false aneurysm and the size of the communication in the tear, different types of endovascular techniques are chosen [12]. Endoluminal grafts require adequate landing zones and are not a safe option in proximity to the coronary ostia and supra-aortic vessels. Septal occluder devices pose a great risk of device migration and embolization and the possibility of a residual leak if they don´t fit the size of the communication in the tear perfectly. Coil embolization requires TAFA with narrow necks but so far is not widely used [13].

Our surgical outcomes were in line with those observed in other studies [1, 9, 10, 13]. Perioperative complications decreased significantly when hypothermic cardiopulmonary bypass was induced prior to surgery [8]. Our outcomes can be attributed to thorough surgical planning, adequate myocardial and cerebral protection, and multi-professional perioperative care.

## Conclusion

Based on our experience, it is recommended that patients who have large TAFA (30mm and above) or fast-growing TAFA (10mm in 6 months or more) should undergo surgery immediately. For small and stable TAFA, regular imaging surveillance is necessary. However, small TAFA that show progression (10mm in 6 months or more) should be treated with either endovascular intervention (such as endoluminal graft implantation, septal occluder implantation, or coil embolization) or redo surgery. If feasible, endovascular intervention is the preferred option.

When considering redo surgery for TAFA that show progression, it is important to weigh the potential risks and benefits. While redo surgery may present a greater burden on the patient, there are strategies to lower possible risks, such as initiating a cardiopulmonary bypass before resternotomy to avoid profuse hemorrhage. However, local repair with PTFE-pledgets may be a superior option if technically possible, as it can shorten the cardiopulmonary bypass and cross-clamp time, thus minimizing the overall risk. Ultimately, the decision should be made on a case-by-case basis with input from the patient's healthcare team.

It's important to have a multidisciplinary team involved in the long-term follow-up of patients who have undergone TAFA surgery. Early detection of any potential issues is key to achieving favorable treatment outcomes. The approach should be tailored to each individual patient to ensure the best possible outcome.

## Abbreviations

CPB: Cardiopulmonary bypass
TAFA: Thoracic aorta false aneurysms
